# Clinical and Technical Challenges of Prosthesis–Patient Mismatch After Transcatheter Aortic Valve Implantation

**DOI:** 10.3389/fcvm.2021.670457

**Published:** 2021-06-04

**Authors:** Pier Pasquale Leone, Fabio Fazzari, Francesco Cannata, Jorge Sanz-Sanchez, Antonio Mangieri, Lorenzo Monti, Ottavia Cozzi, Giulio Giuseppe Stefanini, Renato Bragato, Antonio Colombo, Bernhard Reimers, Damiano Regazzoli

**Affiliations:** ^1^Department of Biomedical Sciences, Humanitas University, Pieve Emanuele, Italy; ^2^Humanitas Research Hospital IRCCS, Rozzano, Italy

**Keywords:** aortic stenosis, prosthesis-patient mismatch, small annuli, TAVI, valve-in-valve

## Abstract

Prosthesis–patient mismatch (PPM) is present when the effective area of a prosthetic valve inserted into a patient is inferior to that of a normal human valve; the hemodynamic consequence of a valve too small compared with the size of the patient's body is the generation of higher than expected transprosthetic gradients. Despite evidence of increased risk of short- and long-term mortality and of structural valve degeneration in patients with PPM after surgical aortic valve replacement, its clinical impact in patients subject to transcatheter aortic valve implantation (TAVI) is yet unclear. We aim to review and update on the definition and incidence of PPM after TAVI, and its prognostic implications in the overall population and in higher-risk subgroups, such as small aortic annuli or valve-in-valve procedures. Last, we will focus on the armamentarium available in order to reduce risk of PPM when planning a TAVI procedure.

## Introduction

Transcatheter aortic valve implantation (TAVI) in patients suffering from aortic stenosis has achieved excellent clinical results in a wide range of patients since its introduction in 2002; originally performed on inoperable patients, it has been progressively implemented in patients at lower risk over the last years, so that the latest American College of Cardiology/American Heart Association guidelines recently surpassed the surgical risk-based decision making for treatment selection in patients with aortic stenosis (1–13).

Despite important improvements in implantation technique and device characteristics, prosthetic valves are still subject to potential failure caused by either structural dysfunction due to permanent intrinsic changes (e.g., wear, cusp rupture, or calcific degeneration) or non-structural dysfunction due to abnormalities not inherent to the prosthesis itself [e.g., paravalvular leak (PVL), malposition, or prosthesis–patient mismatch (PPM)] (14). This review will focus on epidemiology, diagnosis, and clinical impact of PPM after TAVI, and how to prevent it.

## Definition of Prosthesis–Patient Mismatch

The concept of PPM was introduced in 1978, when Rahimtoola first described to consider it when the effective area of the prosthetic valve, after insertion into the patient, is less than that of a normal human valve (15). As a result, a smaller than expected effective orifice area (EOA) might result in higher transvalvular gradients; indeed, the relationship between transprosthetic gradients, flow, and area can be simplified by the following equation:

$$\begin{array}{l} \text{TPG}={\text{Q}}^{2}/\left(\text{k}\times \text{EO}{\text{A}}^{2}\right) \end{array}$$

It shows that the transvalvular pressure gradient (TPG) is directly related to the square of the transvalvular flow (Q) and inversely related to the square of the EOA, while k is a constant. Transvalvular flow depends on cardiac output, which, in turn, is in part determined by the body surface area (BSA); therefore, the EOA needs to be proportionate to the flow requirement for gradients to remain low (16). Vice versa, when the prosthetic valve EOA is too small for the patient's body size, PPM and high transvalvular gradients occur (17–19). Different thresholds of indexed EOA (EOAi), that is the prosthetic EOA divided by patient's BSA (6), have been considered to assess the degree of PPM after aortic valve replacement: an EOAi <0.85 and <0.65 cm^2^/m^2^ are generally considered as thresholds for moderate and severe PPM in the aortic position, respectively (19, 20). In order to avoid overestimation, lower values should be taken into account when evaluating obese patients (body mass index >30 kg/m^2^) (21, 22), i.e., <0.70 cm^2^/m^2^ for moderate PPM and <0.55 or <0.60 cm^2^/m^2^ for severe PPM (23, 24).

Such classification is important, as the prognostic impact of PPM after surgical aortic valve replacement (SAVR) has been related to its severity (25, 26); it is thus key to take into account the methods used to assess it. Indeed, studies in patients undergoing SAVR have mostly used predicted EOAi (EOAip), obtained by dividing the reference value of EOA for the model and size of the prosthetic valve by the patient's BSA, while studies on patients subject to TAVI have used the EOAi measured via the continuity equation by Doppler echocardiography. EOAip obtained from published normal reference values of EOA for each model and size of transcatheter valve has been adopted to determine incidence of PPM after TAVI only in recent reports (27, 28).

## Multimodality Imaging for Assessment of Prosthesis—Patient Mismatch

### Echocardiography

A multi-imaging and multi-parametric approach is key for a comprehensive assessment of transcatheter heart valve (THV) function and an accurate differential diagnosis among different types of valve dysfunction.

**Figure 1:**
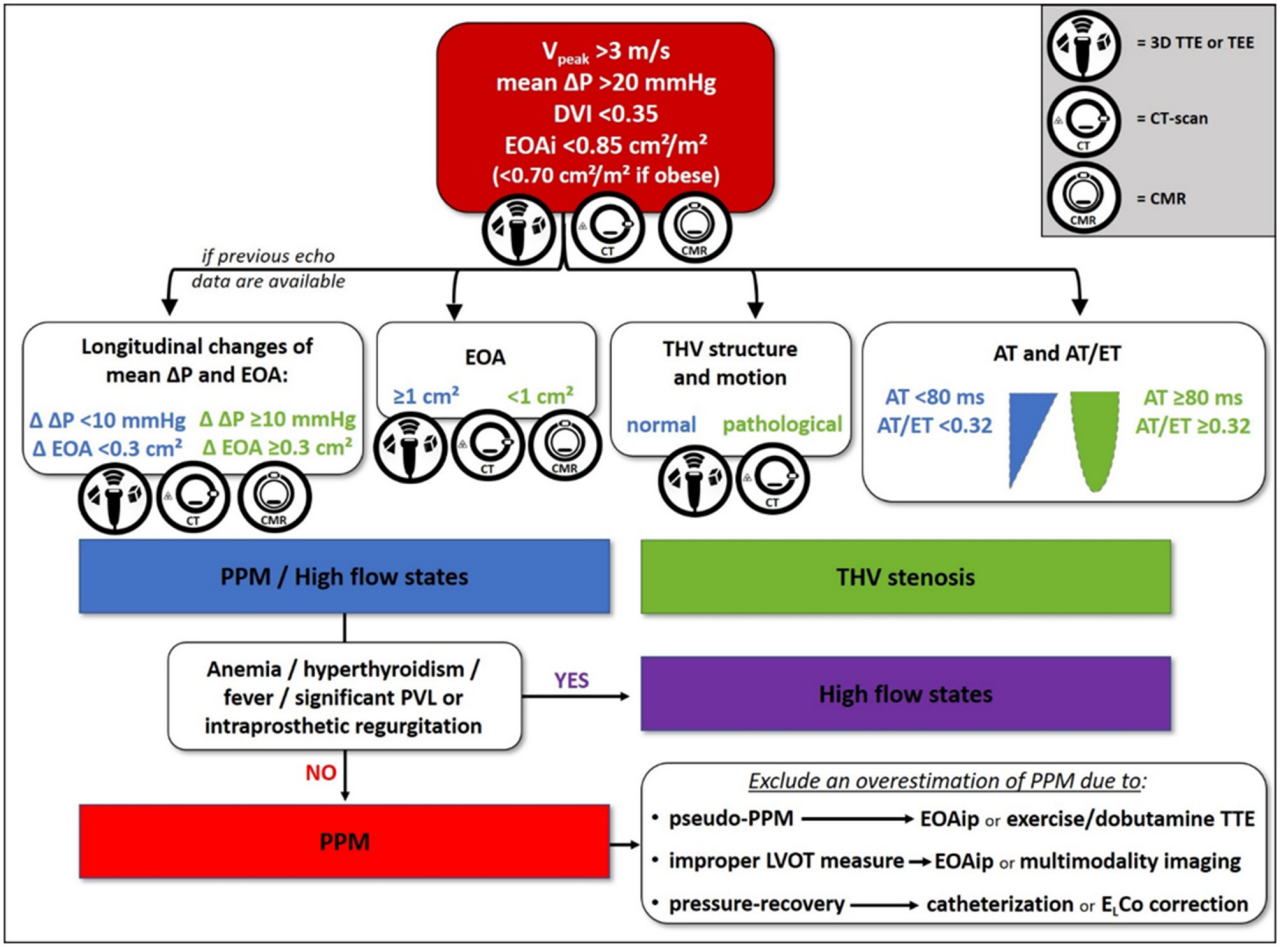
Proposed flowchart for differential diagnosis between high-flow states, transcatheter heart valve (THV) stenosis, and prosthesis-patient mismatch (PPM). 3D, three-dimensional; AT, acceleration time; CMR, cardiac magnetic resonance; CT, computed tomography; Δ, variation; ΔP, pressure gradient; DVI, Doppler velocity index; EOA, effective orifice area; EOAi, indexed effective orifice area; EOAip, predicted indexed effective orifice area; ELCo, energy loss coefficient; ET, ejection time; LVOT, left ventricular outflow tract; PPM, prosthesis-patient mismatch; PVL, paravalvular leak; TEE, transesophageal echocardiography; THV, transcatheter heart valve; TTE, transthoracic echocardiography; V, velocity.

Transthoracic echocardiography (TTE) is the essential tool for initial and longitudinal evaluation of THV function. The echocardiographic finding of increased gradients across a prosthetic aortic valve should activate a diagnostic pathway aiming to exclude the following causes: (1) high-flow hemodynamic conditions, (2) PPM, and (3) acquired stenosis due to either structural valve degeneration or thrombosis (29). We propose a flowchart for differential diagnosis among the above-mentioned causes (Figure 1), mainly based on TTE with additional and complementary aid of three-dimensional TTE or transesophageal echocardiography (TEE), computed tomography (CT), and cardiac magnetic resonance (CMR).

**Figure 2:**
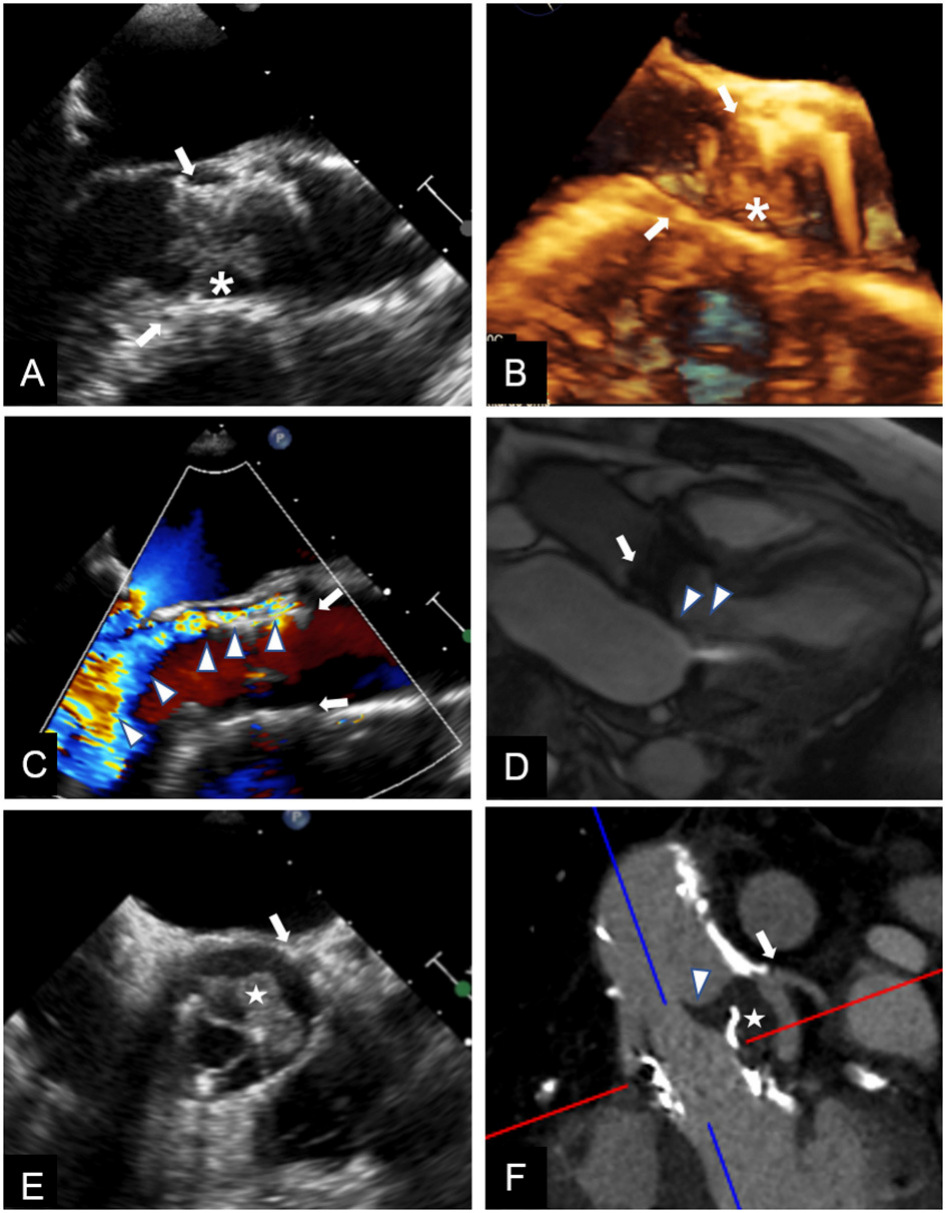
Multimodality imaging for differential diagnosis of high trans-prosthetic gradients in patients with THV. *Case 1*. TEE showing an isoechoic mobile mass (*) adherent to THV (arrows) cusps, causing valve obstruction; blood cultures confirmed infective endocarditis **(A,B)**. *Case 2*. TEE revealing severe paravalvular (arrows) leak (**C**, arrowheads); the regurgitant jet is also shown during CMR cine sequences (**D**, arrowheads), and can be quantified using phase contrast sequences. *Case 3*. TEE in a patient known for valve-in-valve procedure showing an isoechoic mass (**E**, white star) in left coronary sinus, affecting cusp motion (arrowhead). CT **(F)**, confirmed the mass (white star) arising between the two valves, and whose features and density were consistent with thrombus.

First of all, when higher than expected peak aortic jet velocity and gradients through THV are detected, measurement errors such as a contamination of the continuous-wave Doppler signal by mitral regurgitation jet and an excessive gain setting should be excluded (30). Several parameters should be taken into account when evaluating increased transprosthetic gradients. Doppler velocity index is the ratio of velocity–time integrals, which represents an expression of hemodynamic conditions proximal to and through the THV independent of flow and avoids measuring left ventricular outflow tract (LVOT) diameter or area. The qualitative evaluation of THV leaflet morphology and mobility is pivotal to differentiate between vegetation, thrombosis, pannus, a normal THV, and a degenerated one. Shadowing and reverberations due to the struts of THV and suboptimal acoustic windows often limit the diagnostic power of TTE, thus TEE and CT scan may be decisive (Figure 2) (31). The qualitative and semiquantitative assessment of transprosthetic peak velocity may be useful, as a significant stenosis of THV is characterized by a rounded shape of Doppler envelope and a delayed systolic peak, while peak velocity occurs early during systole in normally functioning THV or PPM. The availability of previous echocardiographic data is always valuable, as PPM occurs at the time of TAVI and should be evident since the first post-TAVI echocardiogram, while stenosis may develop any time during the follow-up. The finding of mildly increased transprosthetic gradients during the follow-up with a substantially stable EOA is generally due to an improvement in left ventricular systolic function and a consequent increase in stroke volume (SV).

**Figure 3:**
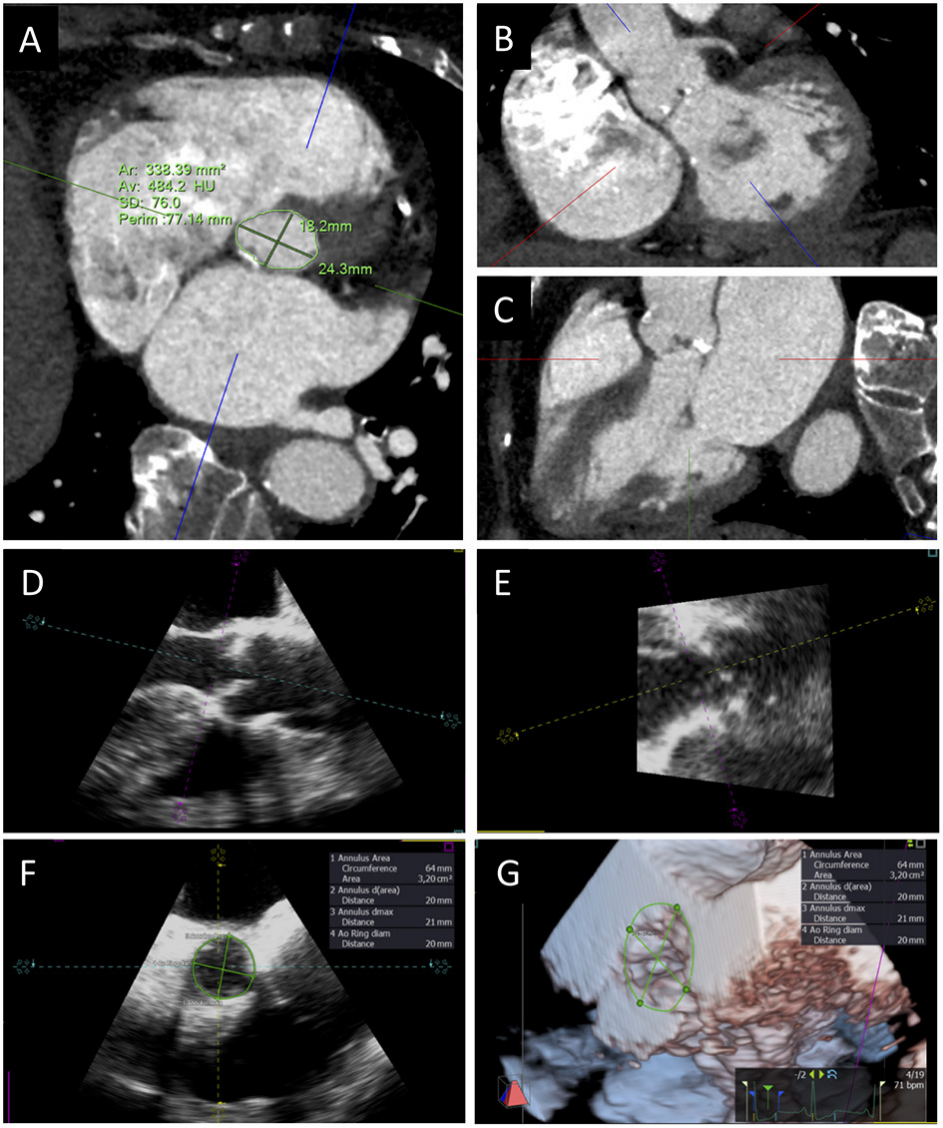
Aortic annulus sizing with CT and 3D TEE. Transverse CT plane aligned at the lowest insertion points of aortic leaflets **(A)** and orthogonal planes oriented along the main axis of LVOT in short **(B)** and long axis **(C)** views. TEE long-axis mid-esophageal view **(D)**, view of the aortic annulus perpendicular to the mid-esophageal view **(E)**, aortic annulus sizing **(F)**, and 3D visualization and measurement of aortic annulus **(G)**.

Finally, EOAi is the fundamental parameter to diagnose PPM and may be measured (EOAim) or, as anticipated, predicted, that is EOAip. Measured EOA is obtained through the continuity equation method, whose numerator is the SV, which may be calculated in different ways. The traditional method to derive SV implies the use of the LVOT diameter and Doppler velocity–time integral (VTI) assessed below the ventricular border of the THV stent (outer-to-outer stent, pre-stent VTI) both measured by two-dimensional TTE (32). However, the use of LVOT diameter is an approximation and introduces a potentially large error, as the LVOT cross-section is normally elliptic and not circular, resulting in constant underestimation of aortic valve area with the continuity equation (33). Nowadays, both three-dimensional echocardiography (TTE or TEE) and CT scan are often used to directly measure LVOT area, which may be then integrated into the continuity equation to better calculate EOAim (Figure 3) (34–36). Indeed, incidence of any PPM among 765 TAVI patients from the PARTNER 2 trial S3i cohort was 24% with EOA estimated by CT compared with 45% with EOA estimated by TTE; of these, only 6 and 9% was graded severe in each group, respectively (34). SV may also be obtained from two- or three-dimensional measurements of ventricular volumes by TTE or CMR (31, 32).

However, EOAim has generally been calculated in most studies and centers by the traditional echocardiographic method (i.e., using the LVOT diameter measurement), which often implies an underestimation of the real size of LVOT area and thus of SV, resulting in an overestimation of the frequency of the PPM (37). Moreover, in low-flow states (SV <35 ml/m^2^), which are not rare, the THV may not fully open, thus, EOAim results are smaller than it would at normal flow with a consequent overestimation of PPM. This latter case is recognized as “pseudo-PPM” in analogy with the concept of “pseudo-severe aortic stenosis” in low-flow low-gradient native aortic stenosis, and could be revealed via contractile flow reserve assessment with dobutamine or exercise echocardiography (37). This may partially explain why, as opposed to the vast majority of evidence supporting a lower risk of PPM after TAVI than SAVR, incidence of severe PPM in STS SAVR (25) and TVT TAVI registries (38) was similar (11 and 12%, respectively).

Ternacle et al. have recently proposed the utilization of EOAip to overcome the issues of pseudo-PPM and technical pitfalls of LVOT area measurement, demonstrating a two- and 10-fold lower incidence of overall and severe PPM, respectively, and a better correlation with transprosthetic gradients when using EOAip as compared with EOAim (27). EOAip represents the “hemodynamic fingerprint” of the prosthetic valve: it is obtained from the published normal reference values of EOA for each model and size of THV (32), is independent of hemodynamic conditions, and is not affected by inter- and intra-observer variability of echocardiographic measurements (39, 40). Also, it can be estimated in all patients, allowing assessment of effect of PPM on periprocedural outcomes, in contrast to the survivorship bias inevitably present when applying EOAim.

Another potential cause of PPM overestimation is the pressure recovery phenomenon, which is responsible for a significant discrepancy between invasive and echocardiographic measurements of transprosthetic mean gradients after TAVI (30, 41). When blood passes through the EOA, its pressure energy is converted to kinetic energy, while after the orifice blood decelerates, and its kinetic energy is either transformed to heat or recovered as pressure energy. The more turbulence occurs after the orifice, the more conversion to heat happens with a consequently reduced difference between net pressure gradient and maximal pressure gradient. The ratio of the valve EOA to the aortic cross-sectional area is the main determinant of the extent of pressure recovery, so that the larger the ratio, the higher the pressure recovery. Different from catheterization, which assesses the net pressure gradient, Doppler echocardiography measures the maximal pressure gradient and cannot take into account the pressure recovery phenomenon. In addition to calculating invasive mean gradients, an alternative to obviate an overestimation of PPM due to pressure recovery, is by adopting the energy loss coefficient, which can be calculated from the echocardiogram using measurements of EOA and aortic cross-sectional area. Interestingly, the extent of pressure recovery may be more important in TAVI than in SAVR, and with the balloon-expandable valve (BEV) than with the self-expandable valve (SEV) (41). Of note, an *in vitro* study assessed the characteristic of hemodynamic flow through an Evolut and a Sapien 3 THV and demonstrated major turbulence with the Evolut THV, which can be due to the different THV constructs, as recently confirmed by Hatoum et al. (42, 43). Therefore, this finding could at least partially explain the known differences between BEV and SEV in terms of echocardiographic mean transprosthetic gradients and measured EOA.

### CT Scan

Aortic annulus evaluation is a crucial step in TAVI procedure planning, and so it is done in order to avoid PPM. Even though an adequate study of the aortic annulus can be made using 3D-transesophageal echocardiography, ECG gated multi-detector row CT is, as of today, the gold standard for pre-operative planning of TAVI (44). CT has the advantages to be non-invasive, less prone to interobserver variability, and safe in patients without contraindications to iodinated contrast.

Many parameters can be derived by CT for annular sizing, including perimeter, area, minimum, mean, maximum, and perimeter-derived diameter. CT confirmation of a small aortic annulus (mean and minimal diameter <23 and ≤ 21 mm, respectively, and/or annular area <400 mm^2^ and/or annular perimeter <72 mm) is key to identify patients at high risk of PPM. Indexed values are even more informative, and should be considered in addition to absolute values. Indeed, a small CT indexed annular area is a reliable pre-procedural predictor of PPM (45), and perimeter-derived annular diameter can be used to identify three groups of indexed aortic annulus size at progressively lower risk of PPM: small indexed annulus size (9–12 mm/m^2^), medium indexed annulus size (12–14 mm/m^2^), and large indexed annulus size (>14 mm/m^2^) (35).

A rigorous measurement of aortic annulus size is pivotal to adequately plan procedures to prevent PPM, especially in patients with smaller annuli. Adequate rate control, breath old, and the use of a last generation scanner are some of the technical aspects needed to obtain adequate CT images of aortic annulus (46). After images acquisition, post-processing multiplanar reconstruction (MPR) is used to visualize the aortic annulus: two orthogonal planes must be oriented along the main axis of LVOT from the LV to the ascending aorta in the short and long axis views; a third transverse plane is aligned at the lowest insertion points of the three aortic cusps to visualize the elliptical shape of the annulus. Once the correct cross-sectional image of the annulus is obtained, maximum and minimum diameters, perimeter, and aortic annulus area can be measured. All measurements should be obtained in mid-systolic phase (generally 30–35% of the RR interval), in order to avoid annulus size underestimation (Figure 3) (47). When available, vendor-specific software can be used to speed up and standardize the computation process (48).

The obtained annulus size can be used pre-operatively to evaluate the EOAip from the normal reference values for THV indexed for patient's BSA (32). As an alternative, CT annular area can be used to directly measure the EOAip, assuming that final THV area and annular area coincide (29). While in SAVR the final EOA is determined by a compromise between surgical manipulation of annulus (debridement of the annulus, removal of the native valve, and eventual enlargement of the aortic root) and surgical valve structure (model, size, and eventual stent presence or sutureless deployment), in TAVI, the main determinant of the final EOA is the annulus itself. Indeed, implantation of a given THV model and size in aortic annuli of different size may yield different EOA, so that use of EOA predicted from aortic annulus area or perimeter measured by CT may be more precise than that predicted from THV model and size for definition of PPM. In this setting, availability of the original measured CT annulus size is helpful for longitudinal assessment of THV function.

It is important to consider also the added value of CT in differential diagnosis of PPM vs. THV stenosis after TAVI (Figure 2). Diagnosis of THV stenosis can be essentially made using TTE as first line diagnostic tool, followed by TEE. Nevertheless, in some cases, differential diagnosis between PPM and THV stenosis is not clear, and multimodality imaging and integration of echocardiographic and CT findings are needed (49–55).

### CMR as Additional Diagnostic Tool

To the best of our knowledge, a systematic assessment of the role of CMR in prevention or diagnosis of PPM has not been investigated yet. However, CMR can be a valid alternative to CT in selected context. CMR has been used both in pre-procedural assessment of patients undergoing TAVI and after TAVI to evaluate valve function ad left ventricle remodeling.

Of note, THV is not a contraindication to CMR, as it is classified as magnetic resonance imaging conditional. The Institute for Magnetic Resonance Safety, Education, and Research web site provides specific indications for each THV model (56). On the other hand, presence of an MRI conditional/safe pacemaker must be verified before referring a patient to a CMR examination. Main disadvantages of CMR are longer scan time, need of greater degree of patient cooperation, and lower image resolution of valve calcifications (indeed, no quantification of calcium is possible). On the other hand, non-contrast CMR can be safely performed in patients with severely impaired renal function or allergy to contrast agents. Annular sizing measurements derived from non-contrast CMR are comparable with those obtained from contrast CT, and can be used to choose valve size (57–59). A hybrid approach with concomitant use of non-contrast CT may overcome the difficulty of CMR to characterize calcifications. The most common non-contrast CMR protocols are (1) steady state free precession (SSFP) cine images along two-chamber, three-chamber, and four-chamber long axis and short axis, followed by two long axis cine images of the aortic root and a stack of cine images acquired orthogonally to the above two planes, covering the entire aortic root; (2) 3D-SSFP navigator-echo and ECG-gated sequence, also called “whole heart” of the aortic root/LVOT. These images can be analyzed in a post-processing manner similar to CT MPR, provided that the mid-systolic phase has been acquired. If a contrast agent can be administered, a multi-step contrast-enhanced magnetic resonance angiography can be utilized. Phase contrast sequences to evaluate valvular function and late gadolinium enhancement sequences to identify myocardial fibrosis can be acquired if clinically indicated (57).

CMR is considered the gold-standard for heart chambers and SV quantification (60). In case of suspected PPM, it can be performed to calculate SV independently of the presence of other concomitant valve disease, like mitral or aortic regurgitation, which can influence echocardiographic assessment of SV. Moreover, CMR showed greater accuracy than echocardiography in quantifying paravalvular regurgitation after TAVI (Figure 2) (61, 62). CMR has been also compared with CT in pre-procedural assessment of valve-in-valve (ViV) in patients with degenerated bioprosthetic valves. In a small analysis of 21 patients, CMR showed good agreement with CT in assessment of aortic geometry and measurement of annulus size. Even if it provided additional information about valve gradients, in some cases the accuracy was limited by the ferromagnetic artifacts of metal strut bioprosthetic valves (63).

In summary, CMR can have an important clinical role in selected TAVI patients: it can be helpful to measure annular size prior to TAVI when the use of contrast medium is contraindicated, identify other causes of hight trans-prosthetic gradients like relevant aortic regurgitation, reclassify PPM or THV stenosis, thanks to a more accurate quantification of SV, and provide useful anatomic and functional information before ViV procedure.

## Epidemiology of Prosthesis–Patient Mismatch

### Surgical Aortic Valve Replacement and Transcatheter Aortic Valve Implantation

Among patients treated with SAVR, the prevalence of overall PPM ranges from 20 to 50% and that of severe PPM ranges from 5 to 25% (29, 64). The prevalence of PPM in patients undergoing TAVI tends to be lower than in surgically treated patients, and is reported between 6 and 46% for moderate PPM and between 0 and 15% for severe PPM (65, 66). Numerous studies, including corollaries of pivotal randomized trials that compared TAVI with SAVR and high number registries reported results on both incidence and clinical impact of PPM after TAVI. The STS/ACC TVT registry reported outcomes after TAVI in 62,125 patients enrolled between 2014 and 2017: moderate and severe PPM were present in 25 and 12% of cases, respectively (Table 1) (72). A recent meta-analysis conducted on 745 patients (399 and 346 undergoing TAVI and SAVR, respectively) described a relative risk reduction of 77% in incidence of PPM when comparing TAVI with surgery (75). Indeed, *post-hoc* analyses of pivotal studies comparing TAVI with SAVR showed that transcatheter bioprostheses result in lower residual gradient, greater valve area, and reduced incidence of PPM (4).

**Table 1:** Selected studies assessing incidence and impact of PPM after TAVI.

**Study**	***N***	**Device**	**Incidence of moderate PPM (%)**	**Incidence of severe PPM (%)**	**Impact of moderate PPM on mortality**	**Impact of severe PPM on mortality**
**Intra-annular BEV**						
PARTNER IA trial (67)^**^	304	Sapien	27	20	HR (95% CI) = 1.10 (0.67–1.80)	HR (95% CI) = 0.58 (0.30–1.13)
PARTNER IA NRCA registry (67)^*^	1,637	Sapien	30	14	HR (95% CI) = 0.94 (0.69–1.29)	HR (95% CI) = 1.20 (0.81–1.78)
PARTNER II S3i cohort (68)^*^	765	Sapien 3	36 18 (CT)	96 (CT)	Any PPM vs. no PPM HR (95% CI) = 0.68 (0.39–1.17) Any PPM vs. no PPM HR (95% CI) = 0.60 (0.30–1.24)	
PARTNER III trial (69)^*^	496	Sapien 3	29	5	HR (95% CI) = 0.95 (0.60–1.50)	HR (95% CI) = 1.31 (0.60–2.86)
**Supra-annular SEV**						
CoreValve US High Risk trial (70)^*^	390	CoreValve	19	7	-	Severe PPM vs. no severe PPM log-rank p = 0.24
Evolut Low Risk trial (71)^*^	722	Evolut R (74%), Evolut PRO (22%), CoreValve (4%)	10	1	-	-
**BEV + SEV**						
STS/TVT ACC registry (72)^*^	62,125	-	25	12	HR (95% CI) = 1.00 (0.93–1.07)	HR (95% CI) = 1.19 (1.09–1.31)
Swiss TAVI registry (73)^*^	448	Sapien, Sapien XT and Sapien 3 (50%)CoreValve and Evolut R (50%)	31 (BEV)27 (SEV)	16 (BEV)7 (SEV)	HR (95% CI) = 0.96 (0.42–2.23) for CV mortality	HR (95% CI) = 1.6 (0.59–4.34) for CV mortality
OCEAN TAVI registry (74)^*^	1,546	Sapien XT (82%), Sapien 3 (9%), CoreValve (9%)	9	1	Any PPM vs. no PPM log-rank p = 0.41	
Registry by Schofer et al. (38)^***^	1,309		23	13	Severe PPM vs. moderate PPM vs. no PPM log-rank p = 0.59	
		Intra-annular BEV (49%)	25	11		HR (95% CI) = 1.89 (1.13–3.16) if LVEF <40%
		Supra-annular SEV (21%)	14	4		
		Intra-annular SEV (12%)	13	24		
		Cusp-fixating SEV (12%)	42	25		
		Infra-annular mechanically-expandable THV (5%)	19	13		
		Non-metallic THV (0.2%)	33	0		
Registry by Ternacle et al. (27)^*^	1,088	Sapien (17%), Sapien XT (39%) and Sapien 3 (27%), CoreValve (4%), Evolut R (13%)	27	17	HR (95% CI) = 1.38 (0.92–2.06)	HR (95% CI) = 1.02 (0.60–1.72)
			10 (predicted)	1 (predicted)	Any PPM vs. no PPM HR (95% CI) = 1.22 (0.67–2.23)	

*BEV, ballon-expandable valve; CI, confidence interval; CT, computed tomography; CV, cardiovascular; HR, hazard ratio; LVEF, left ventricular ejection fraction; PPM, prosthesis-patient mismatch; SEV, self-expandable valve*.

*If not otherwise stated, the reported results are for the following comparisons: moderate PPM vs. no PPM, severe PPM vs. no PPM*.

* *1-year follow-up*.

** *2-year follow-up*.

*** *3-year follow-up*.

### Balloon-Expandable Valves

Regarding trials conducted on transcatheter BEV, incidence of severe PPM in PARTNER cohort A and related registry (67) after implantation of Sapien BEV (Edwards Lifesciences, Inc., Irvine, California) was particularly high, although more frequent (60 vs. 47%) and more often severe (28 vs. 20%) after SAVR than after TAVI (Table 1). Of note, when comparing SAVR with TAVI with Cribier–Edwards (Edwards Lifesciences, Inc., Irvine, California) or Sapien BEV, Clavel et al. reported a higher PPM rate after SAVR with both stented and stentless valves than after TAVI (28 and 20 vs. 6%, *p* = 0.007), thus, highlighting that absence of a sewing ring might not be the only determinant of such favorable hemodynamics after TAVI (76). Similar results were reported in the PARTNER 2A trial, where valve areas were larger and mean transprosthetic gradients were lower in TAVI than in SAVR (10). On the other hand, echocardiographic comparison of SAVR and TAVI with Sapien 3 (Edwards Lifesciences, Irvine, California) in the PARTNER 3 trial showed similar transprosthetic gradients, valve area, rate of severe PPM (4.6 vs. 6.3%, respectively, *p* = 0.30), and LV mass regression in the two arms. This might have been driven by the fact that surgeons implicated in the latter trial were able to implant larger valve sizes, in part due to more aggressive root enlargement; indeed, while reported valve size distribution and hemodynamics of TAVI with Sapien 3 were similar in PARTNER 3 trial and PARTNER 2 Sapien 3 Registry (13, 77), surgical valves ≤ 21 mm were implanted in 44% of patients in PARTNER 2A and only 20% of those in PARTNER 3, so that the hemodynamics of surgical valves was substantially better in the latter trial. Nonetheless, the OCEAN-TAVI registry, conducted in 1,546 Japanese patients with reduced size treated with BEV (1,268 Sapien XT, 139 Sapien 3) and SEV (139 Corevalve), reported a 9.8% incidence of PPM (8.9 and 0.9% for moderate and severe PPM, respectively), and the use of Edwards Sapien 3 was identified as an independent predictor of PPM (74).

### Self-Expandable Valves

The rate of moderate and severe PPM reported in the Evolut Low Risk study in patients undergoing Evolut Pro, Evolut R, and CoreValve (Medtronic, Minneapolis, Minnesota) SEV implantation was lower than reported for BEV; in particular, the incidence of moderate and severe PPM after TAVI and SAVR was 10 and 1% vs. 15 and 4%, respectively (Table 1). This was confirmed in the Swiss TAVI registry, which confronted propensity-score matched patients implanted with SEV (Corevalve, Evolut R, *n* = 224) and BEV (Sapien, Sapien XT, and Sapien 3, *n* = 224): a lower rate of PPM and severe PPM was observed with SEV (33 and 7%) than with BEV (47 and 16%), regardless of aortic annulus size (73). Schofer et al. reported data from 1,309 patients undergoing TAVI with intra-annular BEV, supra-annular SEV, intra-annular SEV, cusp-fixated SEV, mechanically expandable infra-annular valve, and non-metallic valve: the lowest rate of severe PPM was present with supra-annular SEV (4%), whereas the highest rate of severe PPM was detected in patients with self-expandable cusp-fixated (25%) and intra-annular BEV (24%) (38). In a recent further analysis of propensity score-matched patients from the OCEAN-TAVI registry treated with third-generation THV, Evolut R seemed to be superior to Sapien 3 in hemodynamic performance in terms of mean pressure gradient and iEOA (78).

### Small Aortic Annuli

A particular focus on the subgroup of patients with small aortic annuli stems from the fact that these patients showed the greatest benefit in terms of hemodynamics when treated with TAVI vs. SAVR. Indeed, incidence of moderate and severe PPM after TAVI vs. SAVR in this subgroup of patients enrolled in the Evolut Low Risk trial was 7 and 1% vs. 20 and 10%, respectively (71). In this regard, Rodés-Cabau et al. showed in patients included in cohort A of the PARTNER trial and in the parallel non-randomized registry that the hemodynamic advantage of THV is strongly associated with the presence of a small aortic annulus, observing an incidence of severe PPM in this group of almost half compared with that observed in the surgical group (20 vs. 37%, *p* = 0.03). In addition, it is interesting to note that, although the rate of severe PPM after SAVR progressively decreased with increasing aortic annulus size, the rate of severe PPM remained constant regardless of the size of the aortic annulus in patients undergoing TAVI (79).

The hemodynamic advantage of TAVI in this subgroup of patients is particularly evident after SEV implantation. Indeed, supra-annular SEV (Evolut R) had higher EOAi and lower post-procedural mean gradient and PPM than intra-annular BEV (Sapien 3) in the CHOICE-Extend registry (80). Another supra-annular SEV (Acurate Neo, Boston Scientific, Marlborough, Massachussets) similarly resulted in lower gradients and lower rate of severe PPM when compared with Sapien 3 BEV in a multicenter, propensity score-matched study enrolling 246 patients with aortic stenosis and small aortic annuli (81). On the other hand, Portico (St. Jude Medical, Minneapolis, Minnesota) intra-annular SEV showed a rate of PPM similar to Sapien XT intra-annular BEV (10 vs. 13%, *p* = 0.56) in a comparative study of 62 patients treated with 23-mm valves (82), and a higher proportion of moderate PPM than supra-annular SEV in the TAVI-SMALL registry (83). The latter study, a multicenter observational study providing a direct comparison of SEV in a real-world cohort of patients with aortic stenosis and small annuli, provided some insights into the possible mechanism underlying the different hemodynamic behavior of SEV according to prosthetic leaflets position with respect to the native annulus. Indeed, the predisposition to development of higher transvalvular gradients with intra-annular prosthetic leaflets was confirmed in the same study: patients implanted with Acurate Transapical (TA) intra-annular SEV had smaller EOAi and higher mean gradients and PPM than those implanted with Acurate Neo supra-annular SEV (any PPM 66 vs. 11% with Acurate TA and Neo, respectively). Similarly, intra-annular SEV and supra-annular SEV designs showed the highest and lowest rate of severe PPM, respectively, in patients from a single center retrospective study receiving small THV sizes ( ≤ 23 mm) (38). Finally, the favorable hemodynamics of SEV compared with BEV is also tangible when focusing on patients with very small annuli: the mean post-procedural gradient was 10 ± 0.4 mmHg in 175 patients from TAVI-SMALL registry (defined as area- or perimeter-derived diameter <20 mm) implanted with SEV, and 15.4 ± 4.1 and 12.2 ± 4.8 mmHg in 511 patients from OCEAN-TAVI registry (defined as annular area <314 mm^2^) implanted with 20-mm Sapien XT and 23-mm Sapien XT, respectively (83, 84).

These findings were also recently confirmed in Japanese patients with annulus smaller than 330 mm^2^: at least moderate PPM was present post-operatively in 8 and 55% of patients after supra-annular SEV and intra-annular BEV implantation, respectively (*p* = 0.04) (85).

### Valve-in-Valve Procedures

Being SAVR with biological prostheses often preferred over mechanical prosthesis due to lower thrombotic and hemorrhagic complications (86), and given the limited duration of bioprostheses (87, 88), treatment of aged patients with valve degeneration needs to be taken into account (89, 90). Implantation of THV within degenerated bioprostheses (ViV) represents a less invasive alternative to surgery when valve failure occurs (91). However, a small internal diameter and an inelastic stent of surgical bioprostheses often predispose to THV under-expansion during ViV procedures (92), so that high post-procedural gradients and PPM are common after such procedures, with a reported incidence of overall and severe PPM of 37 and 10%, respectively (93). Nonetheless, the type of THV implanted appears to play a role in this setting as well. Indeed, in a recent analysis of ViV procedures performed more than 5 years ago, patients with small surgical valves (internal diameter <20 mm) implanted with supra-annular SEV, when compared with intra-annular BEV, had larger EOA (1.5 ± 0.5 vs. 1.2 ± 0.4 cm^2^, *p* < 0.001) and lower mean gradients (16 ± 9 vs. 21 ± 10 mmHg, *p* < 0.001), notwithstanding a greater prevalence of pre-existing severe PPM in the former group (14 vs. 7%, *p* = 0.022) (94).

Overall, the smaller size of the stent, which the valve is mounted on, the absence of an actual sewing ring occupying annular space, and the systematic oversizing of THV, especially in patients with small aortic annuli, might all be contributing to the superior hemodynamic outcomes with THV compared with surgical valves.

### Predicted Prosthesis–Patient Mismatch

More recently, Ternacle et al. proposed a paradigm shift, and hypothesized that EOAip, routinely used to assess PPM after SAVR, is a more robust parameter to determine the true incidence of PPM following TAVI; thus, they compared incidence and outcomes of PPM according to both measured and predicted EOAi in a retrospective registry including 1,088 patients mostly treated with BEV. The authors found a markedly lower incidence of PPM when assessing it with EOAip (10 and 1% for moderate and severe PPM, respectively) vs. EOAim (27 and 17%, respectively). Indeed, not only did 83% of patients with any degree of measured PPM and 76% of patients with severe measured PPM have no PPM according to normal reference values of EOA, but also incidence of severe measured PPM in the native TAVI sub-cohort (15%) drastically dropped to 0.1% when evaluated with EOAip (Table 1).

Therefore, notwithstanding the favorable hemodynamic profile consistently proven by supra-annular SEV paralleled by larger EOAi when compared with other THV, use of EOAim seems to yield a gross overestimation of PPM incidence after TAVI. The use of EOAip overcomes this limitation, and might provide a more accurate estimation of the true incidence of PPM, which may actually be very rare following TAVI.

## Clinical Impact of Prosthesis–Patient Mismatch

### Surgical Aortic Valve Replacement

The impact of PPM on clinical outcomes in patients undergoing SAVR is known from the first clinical studies conducted in this population, which have shown that PPM is a common and modifiable risk factor leading to poorer hemodynamic valve function and regression of left ventricular (LV) hypertrophy, reduced recovery of coronary flow reserve, and altered coagulation (16, 20, 95). Furthermore, PPM is associated with significant reduction in cardiac index over time, especially in patients with severe PPM, and, in parallel, with significant increase in episodes of congestive heart failure (20, 96). Of utmost importance, it has been shown that it has a strong impact on mortality of patients undergoing SAVR, both in the short- and long-term: in fact, severe PPM, as well as moderate PPM, are associated with an increased risk of mortality at 30 days (the period of greater vulnerability of LV), especially in patients with reduced ejection fraction (97); on the other hand, an increased risk of structural valve degeneration and of rehospitalization for heart failure might explain why such increased mortality risk lasts up to 5–8 years after surgery (24, 98–100). Severe and moderate PPM occurred in 11 and 54% of 59,779 patients ≥65 years of age who underwent isolated SAVR between 2004 and 2014, and was associated with 32 and 8% increase in mortality, respectively (25).

### Transcatheter Aortic Valve Implantation

Despite the above evidence in patients undergoing SAVR, the clinical impact of PPM in patients undergoing TAVI is not entirely clear to date. On one hand, its impact on prosthetic hemodynamic function and on clinical outcomes was progressively more recognized even among patients treated percutaneously (70, 79, 101, 102): PPM was associated with less improvement in functional class, with reduced regression of ventricular mass and diastolic dysfunction, and with increased mortality, especially when severe (67, 103–105). This is concordant with data from the STS/ACC TVT registry, which showed a significant difference at 1 year in terms of mortality in patients with severe, moderate and non-PPM (17.2, 15.6, and 15.9%, respectively, *p* = 0.02), as well as in terms of re-hospitalization for heart failure (14.7, 12.8, and 11.9%, respectively, *p* < 0.0001) (72). While the rate of all-cause mortality was significantly greater in all patients (TAVI and SAVR) with severe PPM compared without severe PPM (21 vs. 12%, *p* = 0.0145) among patients at high surgical risk from CoreValve US High Risk Pivotal Trial (70), other authors report clinical impact of PPM only in selected categories of patients: a high-numerosity prospective single-center registry of TAVI with both BEV and SEV recorded a higher 3-year mortality in patients with severe PPM vs. without PPM only in the subgroup of patients with ejection fraction <40% (68.0 vs. 45.1%, *p* = 0.041) (38).

The importance of forward-flow hemodynamics was stressed by Van Mieghem et al. in a recent analysis on more than 2,000 patients from CoreValve US Pivotal High Risk and SURTAVI intermediate-risk trials: discharge Doppler velocity index (DVI ≤ 0.5 after TAVI was associated with higher 3-year mortality (24 vs. 18%, *p* = 0.025) and mortality or rehospitalization (37 vs. 20%, *p* = 0.007) (68). Similarly, the presence of severe PPM was associated with worse prognosis at 5 years in terms of composite endpoint of cardiovascular death, myocardial infarction, and stroke in a recent analysis on 710 patients (105).

On the other hand, evidence among other available studies is discordant. In a recent meta-analysis conducted on patients treated with both BEV and SEV, although PPM was not rare after TAVI, no significant differences were observed at 30-day (OR 1.51, 95% CI 0.79–2.87), 1-year (OR 1.02, 95% CI 0.96–1.08), and 2-year all-cause mortality (OR 0.99, 95% CI 0.79–1.24) between patients with and without PPM (106). The Swiss TAVI registry did not show significant differences at 1 year in terms of cardiovascular mortality and functional class in patients with or without PPM (Table 1) (73), and 1-year mortality in the PPM group was similar to that in the non-PPM group in the OCEAN-TAVI registry, so that the authors concluded that PPM is not a risk factor for mortality in Asian patients undergoing TAVI (74). A meta-analysis found no statistically significant differences in late mortality between patients with severe PPM and patients without PPM (HR 1.32, 95% CI 0.65–2.67) and between patients with at least moderate PPM and patients without PPM (HR 1.01, 95% CI 0.80–1.27). The authors argue that, while patients without PPM after SAVR exhibit optimal hemodynamic valve performance (i.e., no residual stenosis in the absence of PVL), patients without PPM after TAVI often present PVL which can impair LV mass regression and adversely affect survival, thus, masking the detrimental effect of PPM on patients with no or trace PVL after TAVI (75). Data from PARTNER 1A trial and related registry support this hypothesis: indeed, while severe PPM was associated with increased risk of mortality in the SAVR trial arm, but not in the TAVI trial arm, it had significant impact on survival after TAVI subgroup of patients without post-procedural PVL in the non-randomized continued access registry (67). Nonetheless, when subjects with mild or greater PVL were excluded among TAVI patients from PARTNER 2 S3i cohort, the presence of PPM did not show association with any outcome at 1-year follow-up (34). Also, when patients with and without PPM from the same cohort were confronted, according to EOAi estimated by both TTE and CT, no association between PPM and death or rehospitalization at 1 year was found with either modality, so that such lack of impact on outcomes of PPM after TAVI may not simply derive from an overestimation due to limitations of traditional echocardiographic EOA calculation.

Likewise, in patients undergoing Sapien 3 implantation in the PARTNER 3 trial, 1-year incidence of the primary composite endpoint of mortality, stroke, or rehospitalization rate was similar in patients with and without severe PPM (Table 1), although incidence of severe PPM after TAVI was lower when compared with that reported in the PARTNER 1A trial (5.8 vs. 19.7%) (69).

Thus, while incidence of PPM is generally higher with SAVR than TAVI, and with BEV than with SEV TAVI (67, 70, 76, 107, 108), it is interesting to note that, counterintuitively, its prognostic relevance seems to be more important in SAVR than TAVI, and with SEV than with BEV TAVI (38, 64, 69, 70). The conflicting evidence suggesting that PPM may have a different impact on outcomes according to the type of aortic valve replacement may not derive merely from differences deriving from the approach of valve replacement or from the type of valve, but rather, it likely stems from study and measurement methodological differences, that is, baseline characteristics of enrolled study populations and intrinsic limitations in measurement of parameters needed to estimate EOA after implantation, thus, to identify and quantify PPM, as already described (32). Indeed, several studies reported a more pronounced impact of PPM on survival in younger patients (64); the difference in age between TAVI series and SAVR series (age is often much higher in the former) may thus contribute to such described differences in outcomes. Moreover, while TAVI series have studied mostly the impact of PPM in the short-to-medium term (only few studies reported outcomes at 5 years as of today) (67, 70, 72, 105), SAVR series have assessed its impact on long-term survival (up to 10 years) (25, 39, 64).

In addition, numerous studies did not adjust cutoff values of EOAi in obese patients, so that overestimation of PPM incidence and underestimation of its clinical impact may have occurred.

### Small Aortic Annuli

In this setting, patients with small aortic annuli may be worth of particular attention: although both residual PVL and PPM have been identified as predictors of worst outcome (109), their prognostic role is under discussion, as the relative contribution of each is unclear in this subset of patients. The fact that incidence of significant PVL appears to be lower in patients with smaller aortic annuli (20, 67, 79), as also suggested by results from TAVI-SMALL registry (83), might, at least in part, explain why better survival was recorded after TAVI than after SAVR in patients with small annuli from cohort A of the PARTNER trial, while an opposite trend was observed in patients with large annuli.

### Valve-in-Valve Procedures

In the VIVID (Valve-in-Valve International Data) registry, the presence of small prostheses was associated with increased mortality after ViV implantation at 1 year (110), and up to 8 years (94). In this setting, pre-existing PPM was strongly and independently associated with increased risk for 1-year mortality (OR 1.88; 95% CI: 1.07–3.28) (111). Interestingly, a recent long-term analysis of the VIVID registry found intra-annular BEV utilization among correlates of all-cause reintervention, in addition to pre-existing PPM, malposition, and age (94). Thus, not only do size of the original failed valve and pre-existing PPM seem to impact mortality, but also the type of THV may influence the need for reintervention after aortic ViV, so that operator decisions both during the original bioprosthetic valve implantation and during ViV procedures may affect clinical outcomes.

### Predicted Prosthesis–Patient Mismatch

Finally, the recent contribution by Ternacle et al. sheds some light into the relationship of PPM with outcomes according to the definition taken into consideration. Indeed, while severe predicted PPM had stronger association with high post-procedural gradients compared with severe measured PPM (64 vs. 18%, *p* < 0.001), neither one of them was associated with all-cause or cardiovascular mortality at 1 year, and up to 8 years, in patients with native or ViV TAVI (Table 1). The extent of pressure recovery may be even more represented in ViV procedures (112), and could at least in part explain the contradiction between the important clinical and functional benefit of this procedure and the very high reported rate of severe PPM (113). Indeed, in the study by Ternacle et al., patients with severe PPM (who all belonged to the ViV subcohort) had good clinical outcomes despite high residual gradients, thus may not have exhibited true severe PPM (27). It would not be surprising, since EOAip does not account for pressure recovery, because, first, EOA reference values were derived from native TAVI cohorts, second, pressure recovery is determined by the diameter of ascending aorta and flow patterns within the aorta portion of the valve stent as well.

Finally, while severe PPM seems to be associated with increased short- and mid-term mortality after SEV implantation, its impact on mortality after BEV implantation is more contradictory, with non-randomized data showing a risk of increased mortality, which was not confirmed by randomized trials. Inevitably, definition of PPM and methodological issues (e.g., underestimation of BEV EOA due to pressure recovery) might play a role, and further studies are needed to ascertain it.

## Prevention of Prosthesis–Patient Mismatch

### Patient Selection

In light of the described, although non-univocal, hemodynamic, and clinical impact of PPM (in particular of severe PPM), its prevention in patients undergoing valve replacement needs to be taken into account. However, the preventive strategy should be tailored to underlying clinical characteristics, estimated risk of PPM, and anticipated surgical risk. Indeed, as already introduced, the impact of PPM is not equivalent in all patients, thus underlining the importance of personalized preventive strategies.

In fact, PPM appears to be relatively well-tolerated in elderly and sedentary patients with preserved LV function, while its impact could be harmful on survival and quality of life in young people (<70 years), as well as in patients with impaired ventricular systolic function (ejection fraction <50%), severe left ventricular hypertrophy, concomitant mitral regurgitation, or paradoxical low-flow low-gradient aortic stenosis (21, 39, 97). Younger, physically active patients have higher metabolic demand and higher cardiac output requirements and are therefore more sensitive to the negative impact of PPM on longevity and quality of life. The subgroup of patients most vulnerable to PPM is probably those with pre-existing impairment of ventricular systolic function (97, 99), who can less tolerate an increase in LV afterload. Similarly, residual LV afterload can hinder regression of upstream valvular heart disease after correction of aortic stenosis, e.g., in patients with concomitant significant mitral regurgitation. Indeed, recommendations for surgical bioprosthetic valves include avoidance of severe PPM (EOAi <0.65 cm^2^/m^2^) in every patient, even though this may be not true in obese patients, and avoidance of moderate PPM (EOAi <0.85 cm^2^/m^2^) in patients with LV dysfunction, those with concomitant mitral regurgitation, those undergoing concomitant coronary artery bypass grafting, younger patients, and athletes (64).

**Figure 4:**
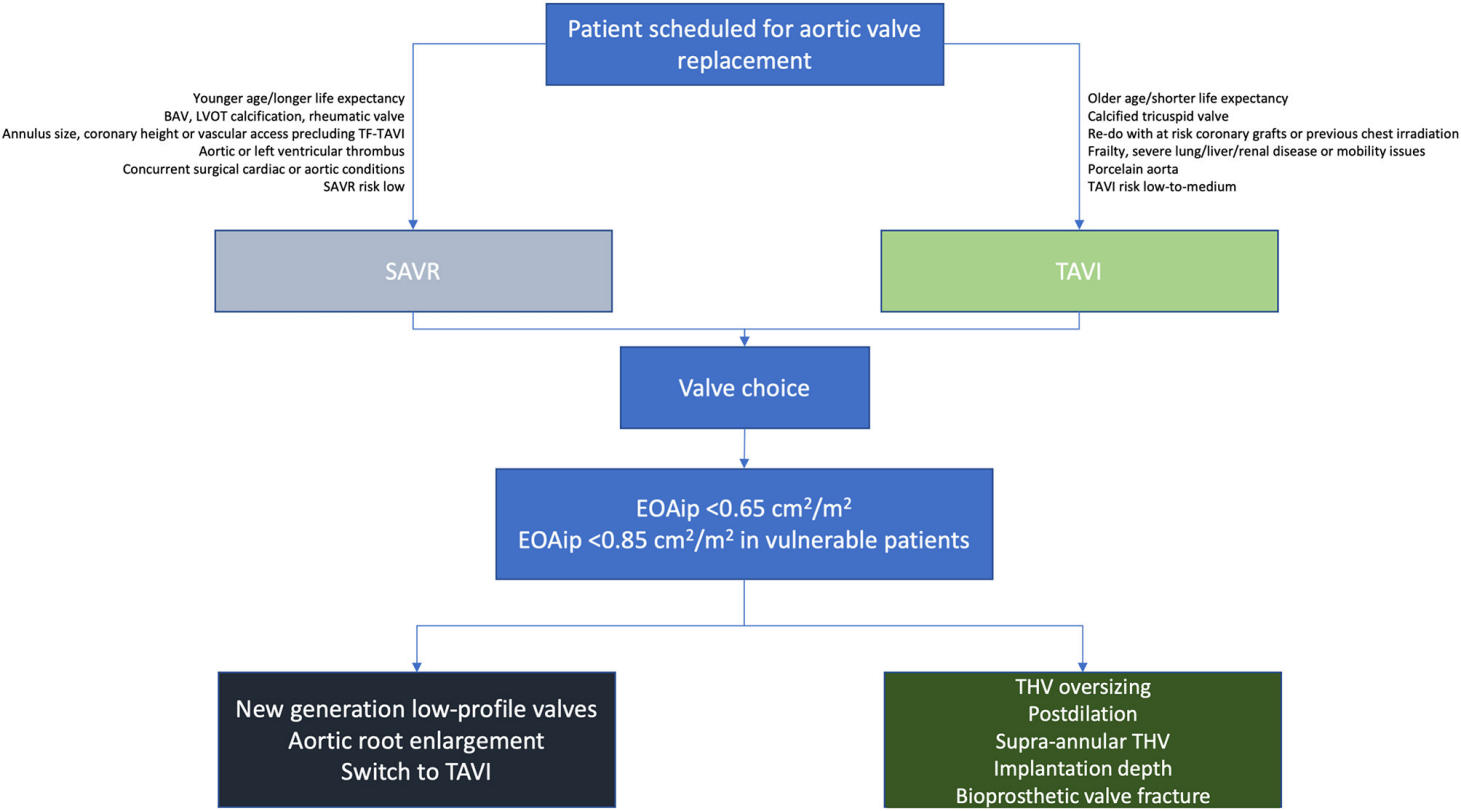
Proposed algorithm for prevention of PPM according to chosen approach of aortic valve replacement. BAV, bicuspid aortic valve; EOAip, predicted indexed effective orifice area; LVOT, left ventricular outflow tract; TF, transfemoral; THV, transcatheter heart valve.

While awaiting results from long-term follow-up of the first randomized trials of TAVI vs. SAVR on patients at low surgical risk, patient selection and PPM prevention procedures have taken into account individual patient surgical risk to guide toward SAVR or TAVI (114). The just released American College of Cardiology guidelines on valvular heart disease somewhat surpass this view, so that surgical risk yielded the floor to other clinical and procedural characteristics for directing toward an approach or the other (Figure 4) (4). Similarly, numerous clinical (younger age, larger BSA), imaging (smaller aortic valve area and annulus area), and procedural (small prosthesis size, use of Sapien 3, no balloon post-dilation, ViV) factors have been associated with increased rate of PPM, and need to be taken into consideration when planning TAVI procedures (72, 74, 75, 115).

### Procedural Planning

SEV have been shown to consistently reduce the incidence of PPM compared with SAVR in annuli of all sizes (101), with greater reduction among patients with small annuli. SEV have also been associated with larger prosthetic valve EOA and lower transprosthetic gradient when compared with BEV (81, 116). This was confirmed by two recently published studies comparing SEV and BEV (73, 105), which suggested that the supra-annular position of prosthetic leaflets may be a key structural feature to yield a favorable hemodynamic profile. In fact, this position allows the avoidance of the superimposed encumbrance of native annulus, native leaflets, stent frame, and prosthetic leaflets, resulting in higher EOA compared with THV with intra-annular architecture. As a matter of fact, the recent PARTNER 3 study, which compared TAVI with Sapien 3 intra-annular BEV and SAVR in patients at low surgical risk, was the first study, among those comparing transcatheter and surgical valve replacement, to show higher gradients and a higher rate of severe PPM after TAVI (13). Although considerations previously cited concerning surgical prosthetic valve size adoption need to be taken into account, the extreme sensitivity of forward hemodynamics to minimal structural modifications employed in between different iterations of the same valve raises some concerns. As what has occurred in the Sapien family of THV with Sapien 3, new reiterations of THV already available on the market have been manufactured focusing on mitigating the incidence of PVL by adding external pericardial “skirt.” Evolut PRO (Medtronic, Minneapolis, Minnesota) is the most recently marketed valve from the CoreValve/Evolut supra-annular SEV family; it is based on the Evolut R platform, with the addition of an external pig pericardium “skirt” (117). Different from the aforementioned intra-annular BEV, no concerns of higher post-procedural gradients were raised from preliminary studies (118). Similarly, recent studies involving the last iteration of Acurate Neo supra-annular SEV, namely, Acurate Neo 2 (Boston Scientific, Marlborough, Massachussets), revealed optimal forward hemodynamics (119). Supra-annular prostheses may, therefore, represent the first choice when treating patients with aortic stenosis and risk factors for PPM. Moreover, given that severe PPM seems to be associated with increased mortality after SEV implantation, the table of expected valve area by either annular measurement or planned THV size might be helpful in estimating expected EOAi, PPM, DVI, and thus predict outcomes. On the other hand, such tables of may be important for follow-up after BEV implantation, in particular when patient-specific baseline data is not available.

In addition to valve type, other planning and procedural aspects may play a protective role with respect to incidence of PPM (Figure 4). While oversizing (between 9 and 15%) was previously reported to be beneficial in SEV implantation (120), a higher degree of perimeter ratio might favor forward hemodynamics in selected patients. Similarly, previous reports have described the protective role of post-dilation in terms of hemodynamics (121–123). Anyhow, such planning and procedural considerations need to be weighed against a possible increase in risk of pacemaker implantation, coronary occlusion, or annular rupture.

### Valve-in-Valve Procedures

As regard ViV procedures, it is clear that PPM prevention starts from the moment of the initial SAVR. As previously described (24), severe PPM is associated with increased mortality and re-hospitalization for heart failure after surgery. In addition, severe PPM can increase mechanical stress of valve leaflets and flow turbulence, which in turn can accelerate structural degeneration of bioprostheses (100, 124, 125), thus anticipating the need for a ViV procedure. Surgeons should therefore aim to implant a bioprosthetic valve with the largest attainable EOA, taking into account patient's body size, by implementing utilization of new generation low-profile, stentless or sutureless bioprostheses, or by performing associated aortic root enlargement to allow implantation of larger bioprostheses. Given that TAVI provides large valve area than same size SAVR, especially in patients with small native aortic annuli (67, 70), TAVI may also be considered as an initial procedure (4). Indeed, ViV in a degenerated THV is associated with lower residual gradients than ViV in a degenerated surgical bioprosthesis (126), and this could be of particular importance in light of a possible Matryoshka doll-like valve-in-valve-in-valve procedure. In patients with pre-existing severe PPM undergoing ViV implantation, supra-annular THV may be preferable (111). The development of new surgical bioprostheses with expandable stent frames and fluoroscopically visible markers may positively impact outcomes following ViV procedures. Moreover, specific implantation targets may optimize hemodynamics after ViV procedures. Indeed, high implantation of both BEV and SEV yielded lower rates of elevated gradients among 292 patients from the VIVID registry. The device position was found to be a stronger predictor of elevated gradients after ViV than the type of device used and surgical valve mechanism of failure, and optimal implantation depths were defined as 0 to 5 mm for CoreValve Evolut and 0 to 2 mm for Sapien XT (127). Finally, an available alternative in this group of patients is to proceed with bioprosthetic valve fracture via pre-dilation, or more commonly post-dilation, with a non-compliant valvuloplasty balloon, thus, facilitating implantation of ViV with SEV or BEV, and potentially reducing residual transvalvular gradients (Figure 4) (128).

In summary, preventive strategies, including prosthetic valve type (129) and size choice and procedural planning, should be considered to avoid or minimize PPM in high-risk patients and in patients vulnerable to PPM. Nowadays, not only is the panorama of THV available on the market gradually expanding but also new reiterations of devices are optimizing profiles of prostheses already on the market, so that pondered choice of the most appropriate valve for the individual patient is a reality.

## Conclusions

PPM is present when the EOA of the implanted prosthetic heart valve is inferior to that of a normal human valve. Multimodality imaging, including echocardiography, CT, and CMR, is of paramount importance in diagnosis and grading PPM after TAVI. PPM might occur more commonly in certain circumstances after TAVI, such as in patients with small aortic annuli or after ViV procedures. Nonetheless, assessment of PPM incidence with EOAim seems to overestimate it, while grading according to EOAip might be more accurate, suggesting this could be a rare event. It is generally more common after SAVR than TAVI, and with intra-annular BEV than with supra-annular SEV TAVI. Its prognostic relevance is not univocal, and seems to be more significant in SAVR than TAVI, and with supra-annular SEV than with intra-annular BEV TAVI. Prevention of this complication is of particular importance in the most vulnerable patients, and careful pre-procedural planning and adequate valve type and size choice are essential.

## Author Contributions

PL, FF, and DR contributed to conception of the review. PL and FF wrote the first draft of the manuscript. FC and JS-S wrote sections of the manuscript. All authors contributed to manuscript revision, read, and approved the submitted version.

## Conflict of Interest

The authors declare that the research was conducted in the absence of any commercial or financial relationships that could be construed as a potential conflict of interest.
